# Effect of Mathematical Modeling and Fitting Procedures on the Assessment of Critical Speed and Its Relationship With Aerobic Fitness Parameters

**DOI:** 10.3389/fphys.2021.613066

**Published:** 2021-05-31

**Authors:** Aurélien Patoz, Nicola Pedrani, Romain Spicher, André Berchtold, Fabio Borrani, Davide Malatesta

**Affiliations:** ^1^Institute of Sport Sciences, University of Lausanne, Lausanne, Switzerland; ^2^Research and Development Department, Volodalen Swiss Sport Lab, Aigle, Switzerland; ^3^Institute of Social Sciences and National Centre of Competence in Research LIVES, University of Lausanne, Lausanne, Switzerland

**Keywords:** running, curve fitting, linear model, hyperbolic model, exponential model, exercise prescription

## Abstract

An accurate estimation of critical speed (CS) is important to accurately define the boundary between heavy and severe intensity domains when prescribing exercise. Hence, our aim was to compare CS estimates obtained by statistically appropriate fitting procedures, i.e., regression analyses that correctly consider the dependent variables of the underlying models. A second aim was to determine the correlations between estimated CS and aerobic fitness parameters, i.e., ventilatory threshold, respiratory compensation point, and maximal rate of oxygen uptake. Sixteen male runners performed a maximal incremental aerobic test and four exhaustive runs at 90, 100, 110, and 120% of the peak speed of the incremental test on a treadmill. Then, two mathematically equivalent formulations (time as function of running speed and distance as function of running speed) of three different mathematical models (two-parameter, three-parameter, and three-parameter exponential) were employed to estimate CS, the distance that can be run above CS (*d′*), and if applicable, the maximal instantaneous running speed (*s*_*max*_). A significant effect of the mathematical model was observed when estimating CS, *d′*, and *s*_*max*_ (*P* < 0.001), but there was no effect of the fitting procedure (*P* > 0.77). The three-parameter model had the best fit quality (smallest Akaike information criterion) of the CS estimates but the highest 90% confidence intervals and combined standard error of estimates (%SEE). The 90% CI and %SEE were similar when comparing the two fitting procedures for a given model. High and very high correlations were obtained between CS and aerobic fitness parameters for the three different models (*r* ≥ 0.77) as well as reasonably small SEE (SEE ≤ 6.8%). However, our results showed no further support for selecting the best mathematical model to estimate critical speed. Nonetheless, we suggest coaches choosing a mathematical model beforehand to define intensity domains and maintaining it over the running seasons.

## Introduction

The prescription of exercise intensity, one of the most important criteria to induce specific adaptations to training (Maclnnis and Gibala, 2017), is often based on the percentage of the maximal rate of oxygen uptake ($\dot{\text{V}}\,{\text{O}}_{2}\,\text{max}$) or maximal heart rate (American College of Sports Medicine, 2000; Burgomaster et al., 2007; Roy et al., 2018). However, among individuals, the lactate threshold, the respiratory compensation point (RCP), and critical power (CP)/speed (CS) were located at different percentages of $\dot{\text{V}}\,{\text{O}}_{2}\,\text{max}$ (Fontana et al., 2015), leading to substantial differences between participants in terms of characteristics of metabolic responses and duration of exercise at a common percentage of the maximum. Therefore, using an exercise prescription based on percentages of maximum values does not guarantee control of exercise intensity (DiMenna and Jones, 2009; Lansley et al., 2011). Instead, a model considering exercise intensity domains for exercise prescription has been recommended (Iannetta et al., 2020). Parameters such as oxygen uptake kinetics (Whipp and Mahler, 1980), ventilatory threshold (VT) (Wasserman et al., 1973), maximum lactate steady-state (Iannetta et al., 2018), and CP/CS (Vanhatalo et al., 2007; Constantini et al., 2014; Jones et al., 2019) can be used to define these various intensity domains.

CP/CS allows defining of the boundary between heavy and severe intensity domains (Jones et al., 2019; Galán-Rioja et al., 2020). Therefore, having an accurate estimation of CP/CS is important. This is obtained by fitting the experimental data to a mathematical model, chosen among several possibilities that differ with respect to their mathematical forms and number of parameters (Monod and Scherrer, 1965; Wilkie, 1980; Moritani et al., 1981; Whipp et al., 1982; Morton, 1986, 1990, 1996; Peronnet and Thibault, 1989). The original linear model formulation was proposed by Monod and Scherrer (1965). This model was applied to cycle ergometry and relates the work performed during an exhaustive bout and its duration through two parameters (two-parameter model): CP (Monod and Scherrer, 1965) or threshold of fatigue (Bigland-Ritchie and Woods, 1984) and the sustainable work of exercise above that metabolic rate (W′) (Monod and Scherrer, 1965). Power has been related to time by dividing the original formulation by the exercise duration (Poole et al., 1986; Gaesser and Wilson, 1988; Housh et al., 1989) while Gaesser et al. (1990) proposed expressing this exercise duration as function of power, which led to the well-known hyperbolic formulation (Morton and Hodgson, 1996). Another model variant, proposed by Morton (2006), expresses the work performed as function of power, since this work (power multiplied by time to exhaustion) is also a dependent variable. However, this model has, to our knowledge, never been used so far.

A straightforward transposition of CP to running was studied by several researchers (Ettema, 1966; Hughson et al., 1984; Housh et al., 1991, 2001; Sid-Ali et al., 1991; McDermott et al., 1993). The CS and distance that can be run above CS (*d′*) are the running analogs of CP and W′, respectively (Hughson et al., 1984; Housh et al., 1991; Pepper et al., 1992; Hill and Ferguson, 1999; Jones and Vanhatalo, 2017). CS is thought to reflect an inherent characteristic of the aerobic energy supply system (Hughson et al., 1984; Gaesser and Wilson, 1988; Poole et al., 1988) and is observed to be correlated with $\dot{\text{V}}\,{\text{O}}_{2}\,\text{max}$ (Hughson et al., 1984; Gaesser and Wilson, 1988; Poole et al., 1988), as well as lactate thresholds (Poole et al., 1988) and RCP (Moritani et al., 1981).

Major shortcomings of the two-parameter model are the assumptions 1) of infinite running speed as time to exhaustion approaches zero, and 2) that at the point of fatigue, *d′* has been completely covered (Gaesser et al., 1995; Morton, 1996). To overcome these limitations, Morton (1996) proposed a three-parameter model including an additional parameter, the maximal instantaneous running speed (*s*_*max*_), and a *d′* that can be only partly covered for a running speed between CS and *s*_*max*_. Alternatively, Hopkins et al. (1989) proposed a three-parameter exponential model based on CS and *s*_*max*_, but where *d′* was replaced by an undefined time constant (τ). The authors reported that their three-parameter exponential model gave better fits than the two-parameter model for inclined treadmill running of short duration (<3 min) (Hopkins et al., 1989). These two- or three-parameter models can be formulated as either distance as function of time, time as function of distance, running speed as function of time, time as function of running speed, distance as function of running speed, and running speed as function of distance, which are mathematically equivalent.

To obtain a statistically appropriate estimation of the model parameters, the correct choice of model formulation and regression analysis should be chosen (Patoz et al., 2021). Such choice is based on the data provided by the experiment and the knowledge of the independent and dependent variables. For the treadmill CS test, running speed is the independent variable while time to exhaustion and distance (implicitly, because it is given by running speed multiplied by time to exhaustion) are the dependent variables. To minimize the error of a model formulation expressing the dependent and independent variables on the vertical and horizontal axes, respectively, the least squares (LS) loss function can be used and requires that the dependent variable be observed with additive error while the independent one would have no additive error (Morton and Hodgson, 1996). Statistical theory has shown that errors in the independent variable are of minor importance, making error minimization in the dependent variable sufficient (Morton and Hodgson, 1996). However, due to heteroscedasticity of the dependent variable (McLellan and Skinner, 1985; Poole et al., 1988; Faude et al., 2017), Morton and Hodgson (1996) suggested to use weighted LS (WLS).

Several researchers have compared the estimation of the parameters provided by the three different models (two-parameter, three-parameter, and three-parameter exponential) and some of their different formulations for cycle ergometry (Gaesser et al., 1995; Bull et al., 2000; Bergstrom et al., 2014) and running on a treadmill (Housh et al., 2001). Significant differences were obtained between the different formulations of the two-parameter model (Gaesser et al., 1995; Bull et al., 2000; Housh et al., 2001; Bergstrom et al., 2014). The three models also differed significantly from one another and the three-parameter model gave the lowest estimation of CP (Gaesser et al., 1995; Bull et al., 2000; Bergstrom et al., 2014) and CS (Housh et al., 2001). However, these studies (Gaesser et al., 1995; Bull et al., 2000; Housh et al., 2001; Bergstrom et al., 2014) did not consider time to exhaustion as the dependent variable, as honestly highlighted by Gaesser et al. (1995). Moreover, these previous studies (Gaesser et al., 1995; Bull et al., 2000; Housh et al., 2001; Bergstrom et al., 2014) are not methodologically exhaustive. Indeed, none of these studies acknowledged heteroscedasticity of the dependent variable.

Hence, the purpose of this study was twofold. First, we compared the estimations of the model parameters obtained by statistically appropriate fitting procedures (the combination of model formulation and regression analysis) applied to the three different models (two-parameter, three-parameter, and three-parameter exponential). We hypothesized that the estimations of CS, *d′*, and *s*_*max*_ would be significantly different between the mathematical models employed, but not between the fitting procedures. We also hypothesized that the three-parameter model would give the lowest estimation of CS, as already observed by Housh et al. (2001) for statistically inappropriate fitting procedures. Second, we determined the correlations between estimated CS and aerobic fitness parameters, i.e., VT, RCP, and $\dot{\text{V}}\,{\text{O}}_{2}\,\text{max}$, as well as the standard error of estimate (SEE) of these relations. We hypothesized that lower quality of the fit [determined by Akaike information criterion (AIC)] would be associated with lower correlations between CS and aerobic fitness parameters and higher SEE.

## Materials and Methods

### Participant Characteristics

Sixteen male runners gave written informed consent to participate in the present experiment (age: 25.6 ± 3.9 years old; height: 1.79 ± 0.05 m; body mass: 69.2 ± 5.3 kg). For study inclusion, participants were required to be in good self-reported general health with no symptoms of cardiovascular disease or major coronary risk factors, no current or recent lower-extremity injury that could prevent them from giving 100% of their capacity during the test or from meeting a certain level of running performance. More specifically, runners were required to have a speed associated with $\dot{\text{V}}\,{\text{O}}_{2}\,\text{max}$ (*s*_$\dot{\text{V}}\,{\text{O}}_{2}$_) greater or equal to 4.44 m/s (16 km/h). The study protocol was approved by the Ethics Committee (CER-VD 2018-01814) and adhered to the latest Declaration of Helsinki of the World Medical Association.

### Experimental Procedure

Each participant completed five experimental sessions interspersed by at least 2 days in the laboratory. All participants were advised to avoid strenuous exercise the day before a test but to maintain their usual training program otherwise. During the first session, participants completed a maximal incremental aerobic test on a treadmill (Arsalis T150—FMT-MED, Louvain-la-Neuve, Belgium). This test consisted of a 10-min warm-up at 2.78 m/s followed by an incremental increase in the running speed of 0.28 m/s every 2 min until exhaustion. Throughout the test, participants breathed into a mask connected to a gas analyzer (Quark, Cosmed, Italy). Pulmonary gas exchange variables [expired minute ventilation ($\dot{\text{V}}\,\text{E}$), oxygen uptake ($\dot{\text{V}}\,{\text{O}}_{2}$), and carbon dioxide output ($\dot{\text{V}}\,{\text{CO}}_{2}$)] were measured breath-by-breath and subsequently averaged over 10-s intervals throughout the test. Before each test, the O_2_ and CO_2_ analyzers were calibrated using room air and known concentrations of calibration gas (16.00% O_2_, 5.02% CO_2_, and the remainder N_2_), and the turbine was calibrated using a 3-L syringe (Hans Rudolph, Germany).

This test was used, first, to determine the peak speed (PS) of the incremental test of each participant. PS is defined as the running speed of the last fully completed increment (*s*_$\dot{\text{V}}\,{\text{O}}_{2}$_) plus the fraction of time spent in the following uncompleted increment (α) multiplied by the running speed increment (Δs = 0.28 m/s) (Kuipers et al., 2003):$P\,\text{S}=s_{\bar{\text{V}}\,{\text{O}}_{2}\,\text{max}}+\alpha \,△\,s.$ Second, the $\dot{\text{V}}\,{\text{O}}_{2}\,\text{max}$ was defined as the highest measured $\dot{\text{V}}\,{\text{O}}_{2}$ value corresponding to (1) a plateau of $\dot{\text{V}}\,{\text{O}}_{2}$ with increased running speed ($\Delta \,\dot{\text{V}}\,{\text{O}}_{2}$ between the last two increments smaller than 50% of the average $\Delta \,\dot{\text{V}}\,{\text{O}}_{2}$ during the submaximal phase of the test) and/or (2) an heart rate greater than 90% of the theoretical maximum heart rate given by 220—age associated with a respiratory quotient greater than 1.1 and a rate of perceived exertion greater than 17. Third, VT and RCP were determined based on gas exchange data and using the method proposed by Wasserman et al. (1973).

The other four tests were performed in a randomized order and consisted of exhaustive runs at a given percentage of the participant’s PS (90, 100, 110, or 120%). These tests were as follows: after a 10-min warm-up at 2.78 m/s and a 5-min rest period, the running speed was increased to a given percentage of PS, and the participant had to maintain the pace until exhaustion. The time to exhaustion was collected for each of the four sessions. No information about the timings or running speed was given to any participant, who were strongly encouraged, during any of the five experimental sessions. All participants were familiar with running on a treadmill.

### Mathematical Modeling

The estimations of CS, *d′*, and *s*_*max*_ were obtained from two different but mathematically equivalent formulations for the three different models. Gaesser et al. (1990) proposed the two-parameter model formulation given by Eq. 1 (non-linear, time-running speed) while Eq. 2 (non-linear, distance-running speed) represents the formulation proposed by Morton (2006). The three-parameter model formulation proposed by Morton (1996) and the inverse of the three-parameter exponential model formulation proposed by Hopkins et al. (1989) are given by Eqs. 3 and 5 (non-linear, time-running speed), respectively, while Eqs. 4 and 6 (non-linear, distance-running speed) represent their distances as a function of running speed formulations.

(1) $$t\,\left(s\right)=\frac{d^{\prime }}{s-\text{CS}}$$

(2) $$d\,\left(s\right)=s\,t\,\left(s\right)=s\,\frac{d^{\prime }}{s-\text{CS}}$$

(3) $$t\,\left(s\right)=\frac{\left(s-s_{\text{max}}\right)\,d^{\prime }}{\left(s-\text{CS}\right)\,\left(\mathrm{CS}-s_{\text{max}}\right)}$$

(4) $$d\,\left(s\right)=s\,t\,\left(s\right)=s\,\frac{\left(s-s_{\text{max}}\right)\,d^{\prime }}{\left(s-\text{CS}\right)\,\left(\mathrm{CS}-s_{\text{max}}\right)}$$

(5) $$t\,\left(s\right)=\tau \,\log\left(\frac{s_{\text{max}}-\text{CS}}{s-\text{CS}}\right)$$

(6) $$d\,\left(s\right)=s\,t\,\left(s\right)=s\,\tau \,\log\left(\frac{s_{\text{max}}-\text{CS}}{s-\text{CS}}\right)$$

*t*, *s*, and *d* stand for time, running speed, and distance, respectively. Of note, distance as a function of running speed was simply given by multiplying time as function of running speed by running speed, i.e., *d*(*s*)=*st*(*s*).

The three-parameter exponential model does not provide a direct estimation of *d′* because the distance that can be run above CS is time-dependent in such a model. Indeed, rearranging the two-parameter model formulation proposed by Whipp et al. (1982) and given by Eq. 7 (i.e., the inverse of Eq. 1)

(7) $$s\,\left(t\right)=\frac{d^{\prime }}{t}+\text{CS}$$

leads to *d*′ = *t*[*s*(*t*)−CS] = *d*(t) − *tCS*. Then, applying this result to the three-parameter exponential model gives an equation where the left-hand side is time-dependent, i.e., *d*′(*t*) = *t*(*s*_max_-CS)*e*^−*t*/τ^. The maximum ($d_{\text{max}}^{\prime }$) of this equation appears where its first derivative is equal to zero, which is at *t* = τ and is given by $d_{\text{max}}^{\prime }$ = τ(*s*_max_-CS)*e*^−1^. This parameter ($d_{\text{max}}^{\prime }$) was used as an estimate of *d*′ for the three-parameter exponential model when comparing the *d′* provided by the different models.

### Data Analysis

Two different fitting procedures were used on the data set obtained for each participant to estimate CS, *d′*, and *s*_*max*_. More specifically, *t*(*s*) and *d*(*s*)using WLS were evaluated. These two fitting procedures are statistically appropriate, i.e., they minimize the error along the axes corresponding to the dependent variables (Vinetti et al., 2020) and should overcome heteroscedasticity Morton and Hodgson (1996). Weights were applied to the corresponding dependent variables, i.e., time to exhaustion in *t*(*s*), and distance in *d*(*s*). Following Morton and Hodgson (1996), weights were set proportional to the inverse of the variance of the dependent variable, where the variance was itself set proportional to the dependent variable. Noteworthy, the model variants *d*(*t*) and *t*(*d*) have not been used. The reason being that in these cases, distance and time to exhaustion should be considered as dependent variables. However, the errors of both variables are correlated, i.e., the error of distance is given by the product of speed and the error of time to exhaustion variable, since speed does not carry any error. This is known as endogeneity and, to the best of our knowledge, there exists no regression method that can handle such case (Antonakis et al., 2014). Error minimization was performed iteratively using the Levenberg-Marquardt algorithm (Levenberg, 1944; Marquardt, 1963). After inspecting residual plots, deviations from homoscedasticity were present for the two fitting procedures applied to the three different models, the three-parameter model with *d*(*s*) and the two-parameter model with *t*(*s*) showing the least and the most heteroscedasticity, respectively (Supplementary Figure 1).

To obtain the $\dot{\text{V}}\,{\text{O}}_{2}$ values at the CS estimates for each participant, first the CS estimates were converted to the times at which these running speeds occurred during the maximal incremental aerobic test assuming a linear relation between running speed and time [i.e., *s*=2.78+0.14*t*, leading to *t*=(*s*−2.78)/0.14, where *t* and *s* stand for time and running speed, respectively]. Then, the $\dot{\text{V}}\,{\text{O}}_{2}$ values at the CS estimates were simply given by placing these corresponding times into the computed linear regression of $\dot{\text{V}}\,{\text{O}}_{2}$ as a function of time recorded during the maximal incremental aerobic test. Data analysis was performed using Python (version 3.7.4, Python Software Foundation^1^).

### Statistical Analysis

Descriptive statistics were expressed as the mean ± standard deviation. The 90% confidence intervals (CI) of CS, *d′* and if applicable, *s*_*max*_, the combined standard error of the estimate (%SEE), i.e., the sum of SEE preliminary transformed to percent units of CS, *d′* and if applicable, *s*_*max*_, and the AIC of the fitting procedure were computed to assess the quality of the fit. For the linear regression of $\dot{\text{V}}\,{\text{O}}_{2}$ as a function of time, its coefficient of determination (*R*^2^) was calculated to examine its accuracy.

After inspecting residual plots, no obvious deviations from homoscedasticity and normality were present. Linear mixed models fitted by restricted maximum likelihood were used to compare CS, *d′*, and *s*_*max*_ obtained from the three mathematical models (two-parameter if applicable, three-parameter, and three-parameter exponential) and two fitting procedures [*t*(*s*) and *d*(*s*)]. The fixed effects included the mathematical models, fitting procedures, and their interaction. The within-subject nature was controlled for by including random effects for participants. The variance explained by the fixed effects over the total expected variance was given by $R_{\text{marginal}}^{2}$ while $R_{\text{conditional}}^{2}$ represented the variance explained by the fixed and random effects together over the total variance (Johnson, 2014). Intraclass correlation coefficients (ICC) of the random effects were computed as the ratios of the variance of the random coefficient divided by the sum of itself and the residual variance. On the basis of commonly used thresholds, poor, moderate, good, and excellent ICCs are given by ICC values <0.5, 0.5–0.75, 0.75–0.90, and ≥0.90, respectively (Koo and Li, 2016). Pairwise *post hoc* comparisons of any significant fixed effects were performed using Holm corrections.

Correlations, 90% CI, SEE (in %), and systematic differences of predicted value (Δ, in %) were computed among the three mathematical models and two fitting procedures with regard to CS, *d′*, and *s*_*max*_ and similarly between CS and aerobic fitness parameters. Data were log transformed as suggested by Hopkins et al. (2009). Correlations were computed using Pearson’s correlation coefficients (*r*). Very high, high, moderate, low, and negligible correlations were given by *r* values of 0.90–1.00, 0.70–0.90, 0.50–0.70, 0.30–0.50, and 0.00–0.30, respectively (Hinkle et al., 2003). Statistical analysis was performed using Python, Jamovi (version 1.0.8, [Computer Software]^2^), and R 3.5.0 (The R Foundation for Statistical Computing, Vienna, Austria) with a level of significance set at *P* ≤ 0.05.

## Results

The variables determined by the incremental test were *s*_$\dot{\text{V}}\,{\text{O}}_{2}$_: 5.05 ± 0.38 m/s, PS: 5.16 ± 0.39 m/s, $\dot{\text{V}}\,{\text{O}}_{2}\,\text{max}$: 63.0 ± 4.9 ml/min/kg, VT: 47.1 ± 3.9 ml/min/kg (74.8 ± 4.1 %$\dot{\text{V}}\,{\text{O}}_{2}\,\text{max}$), and RCP: 56.3 ± 4.8 ml/min/kg (89.3 ± 3.6 %$\dot{\text{V}}\,{\text{O}}_{2}\,\text{max}$). The average *R*^2^ obtained for the linear regression of the $\dot{\text{V}}\,{\text{O}}_{2}$ as a function of time relationship recorded during the maximal incremental aerobic test was 0.94 ± 0.04.

**FIGURE 1:**
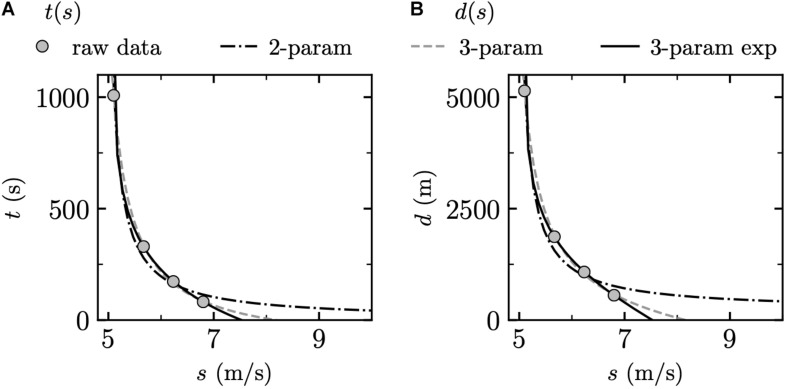
Regression analysis for each of the three mathematical models [two-parameter (2-param), three-parameter (3-param), and three-parameter exponential (3-param exp)] and the two fitting procedures **(A)** *t*(*s*) using weighted least squares (WLS) and **(B)** *d*(*s*) using WLS. *t*: time, *s*: running speed, and *d*: distance.

The regression analysis for one representative participant and for each of the three mathematical models as well as the two fitting procedures [*t*(*s*) and *d*(*s*)] is presented in Figure 1.

Table 1 depicts the time to exhaustion corresponding to the four exhaustive runs performed at 90, 100, 110, and 120% of the participant’s PS.

**TABLE 1:** Means ± standard deviations of the time to exhaustion corresponding to the four exhaustive runs performed at 90, 100, 110, and 120% of the participant’s peak speed (PS).

Running speed (%PS)	90	100	110	120
Time to exhaustion (min)	14.8 ± 2.57	5.94 ± 1.21	2.78 ± 0.78	1.68 ± 0.50

Table 2 depicts CS, *d′*, and *s*_*max*_, together with their corresponding 90% CI, %SEE, and AIC obtained from the three mathematical models and two fitting procedures.

**TABLE 2:** Mean ± standard deviation of the critical speed (CS), distance that can be run above CS (*d′*), and maximal instantaneous running speed (*s*_*max*_), and their corresponding 90% confidence interval (in parenthesis) obtained from the three mathematical models [two-parameter (2-param), three-parameter (3-param), and three-parameter exponential (3-param exp)] and two fitting procedures [*t*(*s*) and *d*(*s*) using weighted least squares] together with the combined standard error of the estimate (%SEE) and the Akaike information criterion (AIC) assessing the quality of the fit.

Mathematical model	Fitting procedure	CS (m/s)	*d*′ (m)	*s*_*max*_ (m/s)	%SEE	AIC
2-param	*t*(*s*)	4.39 ± 0.41 (0.10 ± 0.05)	226.0 ± 57.0 (66.9 ± 26.31)	–	9.8 ± 3.4	32.3 ± 3.4
	*d*(*s*)	4.39 ± 0.40 (0.10 ± 0.05)	222.3 ± 56.0 (65.2 ± 25.1)	–	9.7 ± 3.4	45.8 ± 3.4
3-param	*t*(*s*)	4.12 ± 0.52 (0.27 ± 0.28)	556.9 ± 289.8 (360.6 ± 386.0)	7.72 ± 0.85 (1.50 ± 1.44)	24.8 ± 15.2	24.1 ± 5.3
	*d*(*s*)	4.12 ± 0.52 (0.27 ± 0.27)	546.6 ± 279.1 (352.3 ± 367.7)	7.76 ± 0.88 (1.58 ± 1.59)	25.2 ± 15.3	37.8 ± 5.2
3-param exp	*t*(*s*)	4.55 ± 0.41 (0.12 ± 0.15)	219.5 ± 59.2 (151.6 ± 112.4)	6.96 ± 0.43 (0.55 ± 0.34)	23.9 ± 15.1	24.4 ± 9.0
	*d*(*s*)	4.56 ± 0.41 (0.12 ± 0.15)	217.7 ± 58.0 (150.7 ± 110.0)	6.98 ± 0.43 (0.55 ± 0.35)	24.1 ± 15.2	38.2 ± 8.7

The linear mixed model with random effects explained almost all variance in the data for CS while a large part of variance in the data was still unexplained for *d′* and *s*_*max*_ even with random effects (Table 3). These results were reinforced by the ICC of the random effects, which was excellent for CS but poor and moderate for *d′* and *s*_*max*_, respectively (Table 3).

**TABLE 3:** Percentage of variance explained, fixed effects, and random effects [intraclass correlation coefficient (ICC)] when assessing the effect of the mathematical model and fitting procedure on critical speed (CS), distance that can be run above CS (*d*′), and maximal instantaneous running speed (*s*_*max*_) using a linear mixed model.

	CS	*d*′	*s*_*max*_
**Variance explained**	**%**	**%**	**%**

$R_{\text{marginal}}^{2}$	14.0	45.7	24.5
$R_{\text{conditional}}^{2}$	96.0	72.0	75.6

**Fixed effects**	***P***	***P***	***P***

Mathematical model	**<0.001**	**<0.001**	**<0.001**
Fitting procedure	0.79	0.83	0.77
Mathematical model x fitting procedure interaction	1.00	0.99	0.90

**Random effects**	**–**	**–**	**–**

ICC for intercept	0.95	0.48	0.68

**The variance explained by the fixed effects over the total expected variance was given by*$R_{m\,a\,r\,g\,i\,n\,a\,l}^{2}$*while*$R_{c\,o\,n\,d\,i\,t\,i\,o\,n\,a\,l}^{2}$*represented the variance explained by the fixed and random effects together over the total variance. Statistical significances (P ≤ 0.05) are indicated in bold.**

A significant mathematical model effect was obtained for CS, *d*′, and *s*_*max*_ (*P* < 0.001; Table 3). CS was significantly faster for the three-parameter exponential model compared with CS determined by two- (*P* < 0.001) and three-parameter (*P* < 0.001) models and it was significantly faster for the two- than for the three-parameter model (*P* < 0.001; Table 2). *d′* was significantly lower for the two- and three-parameter exponential model than for the three-parameter model (*P* < 0.001; Table 2). The three-parameter exponential model had a significant slower estimation of *s*_*max*_ than the three-parameter model (*P* < 0.001; Table 2).

No significant fitting procedure effect or significant mathematical model x fitting procedure interaction effect were reported for CS, *d′*, and *s*_*max*_ (*P* ≥ 0.77; Table 3).

On a group level, the average AIC was lower for the three-parameter model for both fitting procedures; however, it was very close to the average AIC for the three-parameter exponential model (Table 2). Note that, because the units of the residual sum of squares error (RSS) depend on the fitting procedure itself, the AICs can be compared between models within a given fitting procedure but not between the two fitting procedures. On an individual level, *t*(*s*) and *d*(*s*) fitting procedures gave the lowest AIC when using the three-parameter model for 12 participants while 4 participants obtained the lowest AIC when using the three-parameter exponential model.

The three-parameter model reported the highest 90% CI as well as the highest %SEE (Table 2). However, %SEE can in general not be compared between the two- and three-parameter models because they do not have the same number of parameters to estimate. Nevertheless, the 90% CI of CS and *d*′ in the three-parameter and three-parameter exponential models were higher than in the two-parameter model, even if expressed in percent units. Therefore, the two models with three parameters carried more error on their estimates than the two-parameter model. The 90% CI and %SEE were similar when comparing the two fitting procedures for a given model (Table 2).

SEE and Δ between CS obtained from the three mathematical models and two fitting procedures ranged from 0.06 to 3.95% and from −0.10 to 0.03%, respectively, while correlations were very high (0.93 ≤ *r* ≤ 1.00; 90% CI: [≥0.84, ≤1.00]) and were all statistically significant (*P* < 0.001). For *d′*, SEE and Δ ranged from 0.58 to 20.2% and from -1.89 to 1.37%, respectively, while correlations were high and very high (0.77 ≤ *r* ≤ 1.00; 90% CI: [≥0.52, ≤1.00]) and statistically significant (*P* < 0.001). For *s*_*max*_, SEE and Δ ranged from 0.62 to 9.09% and from -0.06 to 0.14%, respectively, while correlations were moderate to very high (0.67 ≤ *r* ≤ 1.00; 90% CI: [≥0.34, ≤1.00]) and statistically significant (*P* ≤ 0.004).

The $\dot{\text{V}}\,{\text{O}}_{2}$ at the CS estimates expressed as a percentage of $\dot{\text{V}}\,{\text{O}}_{2}\,\text{max}$ as well as the CS expressed as a percentage of *s*_$\dot{\text{V}}\,{\text{O}}_{2}$_ for the three mathematical models and two fitting procedures are given in Table 4. The $\dot{\text{V}}\,{\text{O}}_{2}$ corresponding to the CS estimates were based on linear regression, therefore, the significant differences between $\dot{\text{V}}\,{\text{O}}_{2}$ values were the same as those for the CV estimates (Table 2; Housh et al., 2001).

**TABLE 4:** Oxygen uptake [$\dot{\text{V}}\,{\text{O}}_{2}$; expressed as a percentage of maximal rate of oxygen uptake ($\dot{\text{V}}\,{\text{O}}_{2}\,\text{max}$)] at the critical speed (CS) estimates as well as CS [expressed as a percentage of speed associated with $\dot{\text{V}}\,{\text{O}}_{2}\,\text{max}$ ($s_{\bar{V}\,O_{2}\,m\,a\,x}$)] for the three mathematical models [two-parameter (2-param), three-parameter (3-param), and three-parameter exponential (3-param exp)] and the two fitting procedures [*t*(*s*) and *d*(*s*) using weighted least squares].

	2-param		3-param		3-param exp	
	*t*(*s*)	*d*(*s*)	*t*(*s*)	*d*(*s*)	*t*(*s*)	*d*(*s*)
$\dot{\text{V}}\,{\text{O}}_{2}$ (%$\dot{\text{V}}\,{\text{O}}_{2}\,\text{max}$)	88.2 ± 4.4	88.3 ± 4.4	83.1 ± 6.7	83.2 ± 6.6	91.3 ± 4.1	91.4 ± 4.1
CS (%*s*_$\dot{\text{V}}\,{\text{O}}_{2}$_)	86.7 ± 2.5	86.8 ± 2.5	81.3 ± 6.2	81.4 ± 6.1	90.1 ± 2.9	90.1 ± 2.8

Correlations, 90% CI, SEE, and Δ between CS and aerobic fitness parameters are given in Table 5. Correlations were high and very high, and all statistically significant (*P* ≤ 0.001).

**TABLE 5:** Pearson’s correlations coefficients (*r*) together with their corresponding 90% confidence intervals (CI), standard error of estimate (SEE, in %), and systematic differences of predicted value (Δ, in %) between critical speed (CS) obtained from the three mathematical models [two-parameter (2-param), three-parameter (3-param), and three-parameter exponential (3-param exp)] and two fitting procedures [*t*(*s*) and *d*(*s*) using weighted least squares] and aerobic fitness parameters [ventilatory threshold and respiratory compensation point (VT and RCP), and maximal rate of oxygen uptake ($\dot{\text{V}}\,{\text{O}}_{2}\,\text{max}$)].

		2-param		3-param		3-param exp	
		*t*(*s*)	*d*(*s*)	*t*(*s*)	*d*(*s*)	*t*(*s*)	*d*(*s*)
VT	*r*	0.85	0.85	0.77	0.77	0.83	0.83
	CI	0.66–0.94	0.66–0.94	0.50–0.90	0.51–0.90	0.63–0.93	0.63–0.93
	*P*	**<0.001**	**<0.001**	**<0.001**	**0.001**	**<0.001**	**<0.001**
	SEE	4.37	4.36	5.50	5.43	4.70	4.67
	*Δ*	–0.13	0.05	–1.46	2.40	1.07	–0.93
RCP	*r*	0.90	0.90	0.77	0.78	0.88	0.88
	CI	0.77–0.96	0.76–0.96	0.52–0.90	0.53–0.91	0.73–0.95	0.73–0.95
	*P*	**<0.001**	**<0.001**	**<0.001**	**0.001**	**<0.001**	**<0.001**
	SEE	3.90	3.88	5.82	5.73	4.27	4.23
	*Δ*	0.01	–0.01	1.99	–2.56	–1.95	2.61
$\dot{\text{V}}\,{\text{O}}_{2}\,\text{max}$	*r*	0.91	0.90	0.85	0.85	0.91	0.91
	CI	0.79–0.96	0.78–0.96	0.66–0.94	0.66–0.94	0.79–0.96	0.80–0.97
	*P*	**<0.001**	**<0.001**	**<0.001**	**<0.001**	**<0.001**	**<0.001**
	SEE	3.63	6.84	4.49	4.43	3.40	3.38
	Δ	0.27	–0.01	–1.38	–2.56	0.40	0.33

**Data were log transformed as suggested by Hopkins et al. (2009). Statistical significance of the correlations (P ≤ 0.05) are indicated in bold.**

## Discussion

Conventional statistical approaches demonstrated a significant effect of the mathematical model when estimating CS, *d′*, and *s*_*max*_, but no significant effect of the fitting procedure. These results validated our first hypothesis that the estimates of CS, *d′*, and *s*_*max*_ would be significantly different between mathematical models employed, but not between fitting procedures. Moreover, the three-parameter model gave the lowest estimation of CS, in accordance with our first hypothesis. Lower SEE and higher correlations between aerobic fitness parameters and CS estimated using a given mathematical model and fitting procedure were not necessarily associated with a lower AIC for these models and procedures, which refuted our second hypothesis.

The linear mixed model showed interindividual differences in CS, *d′*, and s_*max*_, as depicted by the larger $R_{\text{conditional}}^{2}$ than $R_{\text{marginal}}^{2}$ (Table 3), but with a higher impact for CS than for *d′* and s_*max*_, as depicted by the excellent ICC of the random effects for CS but poor and moderate ICCs for *d′* and *s*_*max*_, respectively (Table 3). In addition, a large part of the variance was still unexplained for *d′* and *s*_*max*_ ($R_{\text{conditional}}^{2}$ ≤ 72.0%; Table 3). Therefore, CS could be well estimated by using the mathematical model and fitting procedure, but this is not the case for *d′* and *s*_*max*_. Furthermore, the high and very high between-model correlations (*r* ≥ 0.93) obtained for CS suggest that the estimation of CS provided by each model qualitatively represents the same, as already pointed out by Gaesser et al. (1995). By contrast, some between-model correlations were high and moderate for *d′* and *s*_*max*_, respectively (*r* ≥ 0.67), suggesting less link between estimations of *d′* and *s*_*max*_ from the different regression analyses. Overall, the regression analyses provided more robust estimates of CS than of *d′* and *s*_*max*_.

The three-parameter model gave the lowest AIC on a group level as well as for 75% of the participants for both *t*(*s*) and *d*(*s*) fitting procedures. Nevertheless, the AICs of both three-parameter models were very close to one another (Table 2). The AICs for the two-parameter model were 34% and 21% higher than the three-parameter model ones for *t*(*s*) and *d*(*s*), respectively. Therefore, the two-parameter model gave the lowest quality of the fit, while the three-parameter model seemed to be the most accurate one for both fitting procedures even though it was only slightly better than the three-parameter exponential model. These observations contradict previous findings that obtained similar *R*^2^ values between different mathematical models (Gaesser et al., 1995; Bull et al., 2000; Housh et al., 2001; Bergstrom et al., 2014) [except for a two-parameter linear model expressing power as function of 1/time (Gaesser et al., 1995; Bull et al., 2000)]; this might be explained several ways. First, comparing the accuracy of regression analyses for models based on a different number of parameters (e.g., two vs. three parameters) requires an adjusted *R*^2^ to normalize with respect to the number of parameters within the model. However, these studies (Gaesser et al., 1995; Bull et al., 2000; Housh et al., 2001; Bergstrom et al., 2014) did not mention such usage. Second, *R*^2^ was shown to be an unfavorable measure to describe the validity of a non-linear regression (e.g., both model formulations of the three-parameter model) (Spiess and Neumeyer, 2010) and when using weights in the regression analysis (Willet and Singer, 1988). Therefore, one remaining possibility to compare the quality of the fit of different mathematical models is to use RSS or a parameter that depends on it such as AIC. However, the units of RSS (and thus AIC) being dependent on the fitting procedure (i.e., on the choice of model formulation and axes on which the errors are minimized), the AICs of the various fitting procedures cannot be compared, i.e., AIC of *t*(*s*) cannot be compared to the one of *d*(*s*).

Another option to compare the quality of the fit of different mathematical models is to use %SEE (Triska et al., 2021). However, as already mentioned, %SEE depends on the number of parameters to estimate and is therefore not optimal to compare two- and three-parameter models. Nevertheless, in our case, the 90% CI of CS and *d*′ in the three-parameter and three-parameter exponential models were higher than in the two-parameter model, even if expressed in percent units. Therefore, the two-parameter model gave the lowest %SEE (9.7%) and SEE for CS (0.7%) and *d*′ (9%), while the three-parameter gave the highest %SEE (25%; CS: 2.2%, *d*′:17%, and *s*_*max*_: 5.8%), but only 1% higher than the three-parameter exponential model (24%; CS: 0.8%, *d*′:20.8%, and *s*_*max*_:2.4%). Based on %SEE, the three-parameter model seemed to be the least accurate model, which is in contradiction with the results based on AIC.

CS was thought to reflect an inherent characteristic of the aerobic energy supply system (Hughson et al., 1984; Gaesser and Wilson, 1988; Poole et al., 1988). Such a characteristic is supported by the small SEE and high and very high correlations obtained between CS and aerobic fitness parameters such as VT, RCP, and $\dot{\text{V}}\,{\text{O}}_{2}\,\text{max}$ (SEE ≤ 6.84; *r* ≥ 0.77; Table 5). These results additionally confirm previous observations that showed that CS correlated with $\dot{\text{V}}\,{\text{O}}_{2}\,\text{max}$ (Hughson et al., 1984; Gaesser and Wilson, 1988; Poole et al., 1988) and RCP (Moritani et al., 1981). However, the three-parameter model reported the highest SEE [if we do not consider SEE for the two-parameter model and *d*(*s*)] and smallest correlations, which were associated with the largest 90% CI (4.43 ≤ SEE ≤ 5.82; 0.77 ≤ *r* ≤ 0.85; Table 5). This is in line with the fact that the three-parameter model reported the highest %SEE (Table 2). Nonetheless, SEE and correlations were still small and high, respectively, for this model.

The linear mixed model provided a significant effect of the mathematical model when estimating CS, *d′*, and *s*_*max*_ (Table 3). These results accord with those of previous observations that depicted considerable differences in the estimation of parameters among different models (Gaesser et al., 1995; Bull et al., 2000; Housh et al., 2001; Bergstrom et al., 2014). The three-parameter model provided the lowest estimation of CS on a group level (Table 2) as well as on an individual level. CS estimated using the three-parameter model were 6% and 9% smaller than when using the two-parameter and three-parameter exponential models, respectively. The two-parameter model was shown to produce overestimated CS (Pepper et al., 1992). The authors observed that the time to exhaustion at a running speed set at CS estimated by the two-parameter model was much smaller than expected. Indeed, participants were able to run only 16 min instead of a theoretically indefinite time. Because CS predicted by the three-parameter exponential model was faster than CS predicted by the two-parameter model (+3%; Table 2), we could conclude that the three-parameter exponential model also produced overestimated CS.

The observed between model differences for the CS estimates (up to 0.44 m/s, Table 2) are not negligible and would certainly have an impact when prescribing a training session based on exercise intensity. Therefore, we encourage coaches prescribing exercise based on critical intensity to choose a mathematical model beforehand to estimate CS and maintain it over the running seasons, so that CS is always estimated in the same way. Moreover, even though the estimated CS should be a very good approximation of the critical intensity but not the critical intensity *per se*, we suggest to physiologically verify that the estimated CS represents the upper boundary of sustainable exercise. In addition, coaches should not hesitate to make small adjustments based on the observed performance. Moreover, given the day-to-day variation of human performance and the CI of the estimated CS, i.e., about 5% of its value (Table 2), it would be justified to prescribe exercise intensity outside these confidence limits to avoid being in the phase transition between the heavy and severe intensity domains (Anderson et al., 2019).

Jones and Vanhatalo (2017) found that CS occurred at 70–90% of $\dot{\text{V}}\,{\text{O}}_{2}\,\text{max}$, depending on training status (the higher the training status, the higher the CS in $\%\dot{\text{V}}{\text{O}}_{2}\text{max}$). In the present study, the $\dot{\text{V}}\,{\text{O}}_{2}$ at the CS estimates for the three-parameter model were close to the middle of the range defined by Jones and Vanhatalo (2017) (83%; Table 4), while the $\dot{\text{V}}\,{\text{O}}_{2}$ at the CS estimates for the two-parameter and three-parameter exponential models were in the higher end of the range (≥88.2 %$\dot{\text{V}}\,{\text{O}}_{2}\,\text{max}$; Table 4). Higher $\dot{\text{V}}\,{\text{O}}_{2}$ at the CS estimates were already reported by Housh et al. (2001) for the two-parameter and three-parameter exponential models (≥94 %$\dot{\text{V}}\,{\text{O}}_{2}\,\text{max}$) than for the three-parameter model (89 %$\dot{\text{V}}\,{\text{O}}_{2}\,\text{max}$). These authors even reported $\dot{\text{V}}\,{\text{O}}_{2}$ at the CS estimates that exceeded $\dot{\text{V}}\,{\text{O}}_{2}\,\text{max}$ for the exponential model (105 %$\dot{\text{V}}\,{\text{O}}_{2}\,\text{max}$). In the present study, CS corresponded to 81, 87, and 90 %*s*_$\dot{\text{V}}\,{\text{O}}_{2}$_ for the three-parameter, two-parameter, and three-parameter exponential models, respectively. Billat et al. (1995) observed that CS corresponded to 86% of *s*_$\dot{\text{V}}\,{\text{O}}_{2}$_ for runners having 75 ml/min/kg of $\dot{\text{V}}\,{\text{O}}_{2}\,\text{max}$ and 6.22 m/s of *s*_$\dot{\text{V}}\,{\text{O}}_{2}$_. These values were higher than those of the participants of this study (+16 and +19%, respectively). Therefore, we could speculate that CS estimated by the three-parameter model (81 %*s*_$\dot{\text{V}}\,{\text{O}}_{2}$_) is closer to reality than CS estimated by the other two models (≥87 %*s*_$\dot{\text{V}}\,{\text{O}}_{2}$_). Both arguments reinforce the idea that both two-parameter and three-parameter exponential models overestimate CS. In any case, a future study involving exhaustive runs below, at, and above CS whilst assessing oxygen uptake responses to exercise would be needed to quantitatively validate this suggestion.

The estimation of *d′* using the three-parameter model were roughly 2.5 times larger than those from the other two models. These findings are consistent with those of previous studies (Gaesser et al., 1995; Morton, 1996; Bull et al., 2000; Housh et al., 2001; Bergstrom et al., 2014). Morton (1996) suggested that such a model overcomes physiological assumptions of the two-parameter model such as an infinite power when time approaches zero and that at *d′*, the muscular energy reserve is empty. Assuming an *s*_$\dot{\text{V}}\,{\text{O}}_{2}$_ of 6 m/s and a time to exhaustion of ∼5 min at 100% of *s*_$\dot{\text{V}}\,{\text{O}}_{2}$_ (Billat et al., 1995), the corresponding total distance covered is 1,800 m. The anaerobic contribution was shown to represent approximately 10% of the total distance covered, i.e., approximately 200 m (Billat, 2001). Therefore, because Morton (1996) suggested that the three-parameter model allows *d′* to be only partly covered for a running speed between CS and *s*_*max*_, this statement causes the estimate of *d′* that is larger in the three-parameter model than in the other two models to not be unrealistically high. This idea is reinforced by an explanation based on anaerobic energy calculation by Gaesser et al. (1995).

Buchheit and Laursen (2013) found that athletes with similar *s*_$\dot{\text{V}}\,{\text{O}}_{2}$_ to those of the present study had a *s*_*max*_ ranging from 161 to 183 %*s*_$\dot{\text{V}}\,{\text{O}}_{2}$_. Higher level athletes (*s*_$\dot{\text{V}}\,{\text{O}}_{2}$_ = 6.36 m/s) were shown to have a lower relative *s*_*max*_ (149 %*s*_$\dot{\text{V}}\,{\text{O}}_{2}$_) (Sandford and Stellingwerff, 2019). Therefore, the estimation of *s*_*max*_ using the three-parameter exponential model seemed to be unrealistically too small (∼136 %*s*_$\dot{\text{V}}\,{\text{O}}_{2}$_) whereas the one obtained using the three-parameter model seemed closer to reality (∼155 %*s*_$\dot{\text{V}}\,{\text{O}}_{2}$_). Nonetheless, this has to be nuanced by the fact that most of the running trials at 120 %PS gave a time to exhaustion below 2 min, which is below the usual recommendation (Jones and Vanhatalo, 2017) and could have influenced the estimation of the parameters present in the mathematical models. In addition, participants were long distance runners, meaning that they are not accustomed to running at high speeds (i.e., >100 %*s*_$\dot{\text{V}}\,{\text{O}}_{2}$_) and that they actually did not have a high *s*_*max*_. This assumption is supported by the observations of Sandford and Stellingwerff (2019), who showed that a 400-m elite runner (*s*_$\dot{\text{V}}\,{\text{O}}_{2}$_ = 6.23 m/s) had a *s*_*max*_ of 158 %*s*_$\dot{\text{V}}\,{\text{O}}_{2}$_ while a 1,500-m elite runner (*s*_$\dot{\text{V}}\,{\text{O}}_{2}$_ = 6.45 m/s) had a *s*_*max*_ of 141 %*s*_$\dot{\text{V}}\,{\text{O}}_{2}$_.

No significant fitting procedure or mathematical model x fitting procedure interaction effects were reported for the estimations of CS, *d*′, and *s*_*max*_ (Table 3). Gaesser et al. (1995) proposed that differences between the estimation of parameters among models could come from the designation of the dependent and independent variables, the number of parameters in each model, and the choice of model (e.g., two-parameter, three-parameter, or three-parameter exponential). Moreover, two mathematically equivalent model formulations requiring linear vs. non-linear regressions were shown to provide different estimations of their underlying parameters (Colquhoun, 1971). In this study, we observed that using different but statistically appropriate fitting procedures, i.e., that correctly attribute the dependent and independent variables, applied to a given model did not have an impact on the estimations of CS, *d′*, and *s*_*max*_, as long as all the model formulations are non-linear or linear.

Heteroscedasticity of the dependent variable was explicitly depicted by Hinckson and Hopkins (2005) when using usual LS fitting procedure. Indeed, these authors demonstrated systematic and non-uniform deviation from their models by showing the residuals as function of predicted values. In this study, the suggestion made by Morton and Hodgson (1996) to overcome heteroscedasticity, i.e., weights proportional to the inverse of the values of the dependent variable, were applied. However, the absolute weighted residuals as function of predicted values for the two fitting procedures applied to the three different models depicted clear deviations from homoscedasticity (Supplementary Figure 1). Therefore, considering weights in the fitting procedure did not overcome the heteroscedasticity problem. Nonetheless, a future study considering different weighting schemes should be performed in order to observe if a specific weighting scheme, different from the one proposed by Morton and Hodgson (1996), could overcome heteroscedasticity of the dependent variable.

Some limitations to the present study exist and need to be addressed. On the one hand, the participant should complete five experimental sessions interspersed by at least 2 days, which could be slightly unpractical. On the other hand, performing a regression analysis with only four measurement points is already quite few, especially when dealing with heteroscedasticity. Nonetheless, the estimation of CS based on four points is considered as the best practice (Poole et al., 2021). Moreover, there is a well-known large variability in the time to exhaustion during treadmill running at CS (Pepper et al., 1992). Furthermore, due to the proximity between CS and RCP in terms of %$\dot{\text{V}}\,{\text{O}}_{2}\,\text{max}$ (CS: 87.6 %$\dot{\text{V}}\,{\text{O}}_{2}\,\text{max}$; RCP: 89.3 %$\dot{\text{V}}\,{\text{O}}_{2}\,\text{max}$) and the high and very high correlations between them (*r* ≥ 0.85), one could wonder the relevance of CS. However, the recent meta-analysis of Galán-Rioja et al. (2020) showed that CS and RCP are not synonymous. Besides, CS can be estimated using personal best times, which does not require the participant to go to the laboratory (Jones et al., 2019). Finally, a recent study demonstrated that using estimations of CS from raw training data can be sufficient to successfully predict marathon performance and provide useful pacing information (Smyth and Muniz-Pumares, 2020).

To conclude, this study demonstrated that CS, *d′*, and *s*_*max*_ estimated from three different mathematical models (two-parameter, three-parameter, and three-parameter exponential model) differed significantly, but that no difference in the estimation of CS, *d′*, and *s*_*max*_ was reported between different statistically appropriate fitting procedures applied to a given model. Weights did not help overcoming heteroscedasticity of the dependent variable. CS estimates from the three different models were correlated with aerobic fitness parameters, i.e., VT, RCP, and $\dot{\text{V}}\,{\text{O}}_{2}\,\text{max}$. Moreover, small SEE was obtained. The three-parameter model gave the lowest AIC on a group level and the smallest CS estimates. However, the three-parameter model reported the highest %SEE and 90% CI. Therefore, our results showed no further support for selecting the best mathematical model to estimate critical speed. Nevertheless, our results showed that statistically appropriate fitting procedures gave the same estimates for a given model. For these reasons, we suggest coaches choosing a mathematical model with appropriate fitting procedure beforehand to define CS and intensity domains and maintaining it over the running seasons. Moreover, our findings suggest that each CS estimation during season should be physiologically verified and training prescription should be done around CS (±10%) for taking into account CI of its estimation and the day-to-day variation of human performance.

## Data Availability Statement

The raw data supporting the conclusions of this article will be made available by the authors, without undue reservation.

## Ethics Statement

The studies involving human participants were reviewed and approved by CER-VD 2018-01814. The patients/participants provided their written informed consent to participate in this study.

## Author Contributions

FB and DM: conceptualization and supervision. FB, DM, and AP: methodology. FB, DM, RS, and NP: investigation. AP and AB: formal analysis. AP: writing—original draft preparation. AP, AB, FB, and DM: writing—review and editing. All authors contributed to the article and approved the submitted version.

## Conflict of Interest

The authors declare that the research was conducted in the absence of any commercial or financial relationships that could be construed as a potential conflict of interest.

## Footnotes

1 http://www.python.org

2 https://www.jamovi.org
