# Supplementary material for: Effect of Mathematical Modeling and Fitting Procedures on the Assessment of Critical Speed and Its Relationship With Aerobic Fitness Parameters
Source: Front Physiol. 2021 May 31;12:613066. doi: 10.3389/fphys.2021.613066 (PMC8201789; doi:10.3389/fphys.2021.613066)
Supplement: Supplementary file 1 [file Image_1.pdf]

Supplementary Material

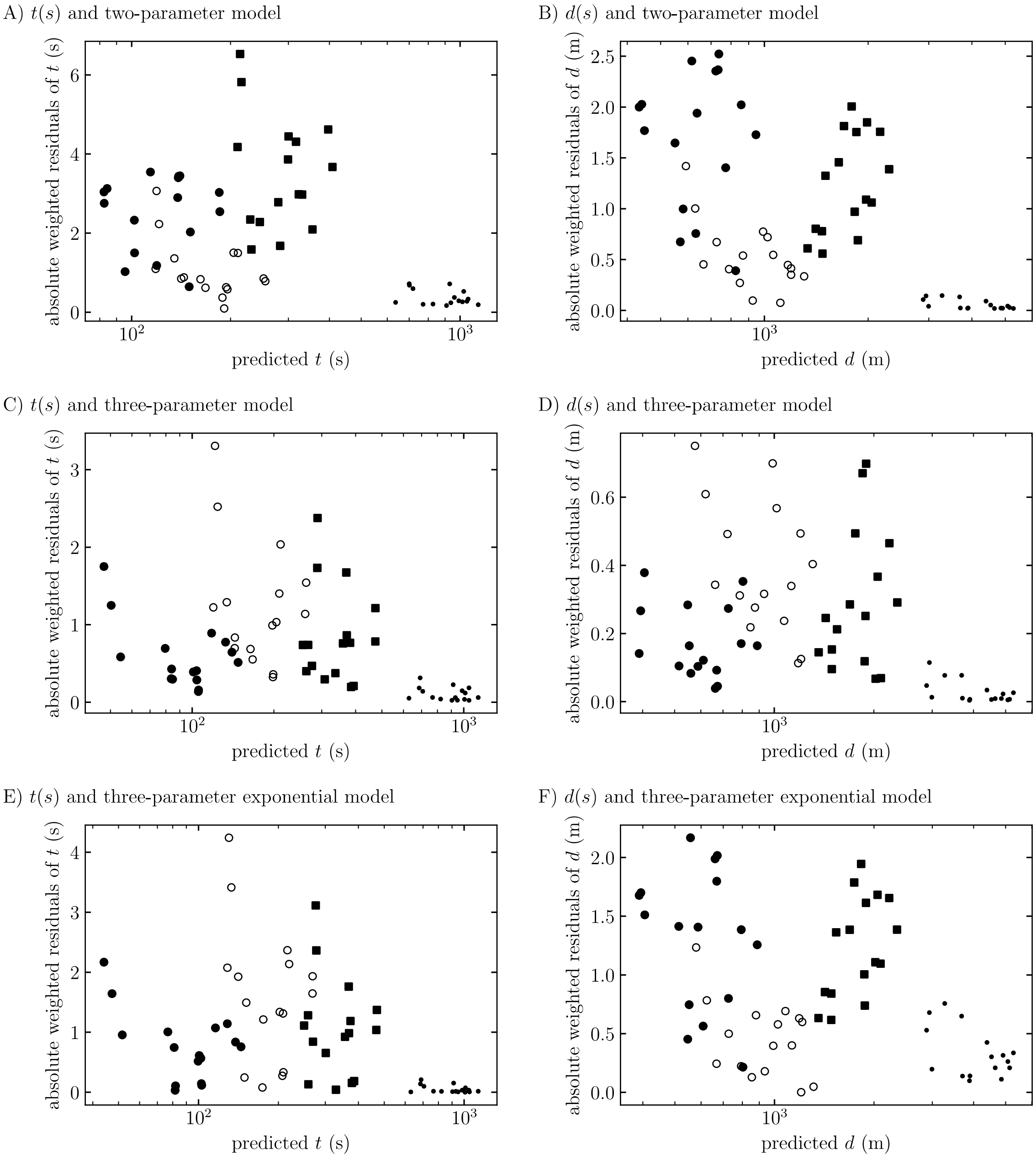

Supplementary Figure 1. Residuals as function of predicted values for (A) *t(s)* and two-parameter model, (B) *d(s)* and two-parameter model, (C) *t(s)* and three-parameter model, (D) *d(s)* and three-parameter model, (E) *t(s)* and three-parameter exponential model, and (F) *d(s)* and three-parameter exponential model. The fitting was performed using weighted least square regression. The four symbols represent the set of four exhaustive runs of each runner: 90% (·), 100% (■), 110% (o), and 120% (•) peak speed of the incremental test.
